# The effect of ROCK-1 activity change on the adhesive and invasive ability of Y79 retinoblastoma cells

**DOI:** 10.1186/1471-2407-14-89

**Published:** 2014-02-14

**Authors:** Jing Wang, Xiao-Hong Liu, Zi-Jian Yang, Bing Xie, Yi-Sheng Zhong

**Affiliations:** 1 Department of Ophthalmology, Ruijin Hospital Affiliated Medical School, Shanghai Jiaotong University, 197 Ruijin No. 2 Road, Shanghai 200025, PR China

**Keywords:** Adhesion, Invasion, Retinoblastoma, ROCK

## Abstract

**Background:**

Retinoblastoma (Rb) is the most common intraocular tumor in childhood worldwide. It is a deadly pediatric eye cancer. The main cause of death in Rb patients is intracranial and systemic metastasis. ROCK is the main downstream effector of Ras-homologous (Rho) family of GTPases which are involved in many cellular functions, such as cell proliferation, invasion and metastasis. Overexpression of ROCK promotes invasion and metastasis of many solid tumors. However, the effect of ROCK in Rb is largely unknown.

**Methods:**

ROCK-1 and ROCK-2 mRNA expression in Y79 cell lines were examined by RT-PCR. Protein expression in the Y79 cell line were examined by western blot analyses. ROCK-1 and ROCK-2 siRNA were transfected into Y79 cells with Lipofectamine 2000. Cell proliferation was evaluated by CCK-8 assay after exposure to ROCK inhibitor (Y-27632). We examined the effect of ROCK inhibitors (Y-27632, ROCK-1 and ROCK-2 siRNA) on Y79 cell adhesive capacity by cell adhesion assay. Cell invasion assay through matrigel was used to study the effect of ROCK inhibitors on Y79 cell invasive capacity.

**Results:**

The expression of mRNA of ROCK-1 was more than that of ROCK-2 in the Y79 cell line. The protein expression levels of ROCK-1 and ROCK-2 were downregulated in the cells transfected with siRNA. Y-27632 treatment didn’t lead to any changes of Y79 cells proliferation. Adhesive ability of Y79 cells was enhanced following Y-27632 or ROCK-1 siRNA treatment. The invasive capacity of Y79 cells showed an inverse relationship with increasing Y-27632 concentration. Invasiveness of Y79 cells also decreased in Y79 cells transfected with ROCK-1 siRNA. However, there was no change in adhesive ability or invasive capacity in Y79 cells transfected with siRNA against ROCK-2.

**Conclusions:**

The findings of this study demonstrate that ROCK-1 protein plays a key role in regulating metastasis and invasion of Y79 cells, suggesting that the ROCK-1 dependent pathway may be a potential target for therapy of Rb.

## Background

Retinoblastoma (Rb) is a deadly pediatric eye cancer. The mortality rate among children diagnosed with Rb is 50% to 70% in the underdeveloped countries, and it is the most common intraocular tumor in childhood worldwide [1]. Children with Rb are at risk for three life-threatening problems, including metastasis of Rb, intracranial neuroblastic malignancy (trilateral Rb), and second primary tumors [2].

The changes of motility are among the initial events of invasion and metastasis. Dynamic reorganization of the actin and tubulin cytoskeleton facilitate cell movement [3,4]. Among the signaling pathways participating in regulating invasion and metastasis of cancer cells, Rho associated kinase (ROCK) signaling pathway plays a key role in the process [5]. To date, two ROCK isoforms have been described, namely ROCK-1 and ROCK-2. They are highly homologous, sharing 65% of the entire in amino acid sequence and 92% of the sequence in their kinase domains [6]. ROCK is the main downstream effector of Ras-homologous (Rho) family of GTPases which are involved in many cellular functions, such as cell proliferation, apoptosis, invasion and metastasis [7]. Overexpression of ROCK promotes invasion and metastasis in many solid tumors, such as hepatocellular, breast and colon cancers [8-11]. Therefore, inhibition of ROCK could be a potential therapeutic approach for these tumors.

Y-27632 is a well-established pharmacological inhibitor displaying a high specificity for ROCK proteins [12]. Y-27632 treatment decreases invasion of cultured melanoma and other tumor cells [13,14]. However, the effect of ROCK inhibition in Rb is largely unknown. In this study, we investigated the effect of ROCK pathway inhibition by using Y-27632 and ROCK siRNA on proliferation and motility of Rb cells.

## Methods

### Cell lines and cell culture

Rb Y79 cell line was obtained from ATCC. RPMI 1640 media and fetal bovine serum (FBS) were purchased from Life Technologies Corporation. Y79 cells were cultured in RPMI 1640 medium supplemented with 15% heat-inactivated FBS, 0.1% ciprofloxacin, 2 mM L-glutamine, 1 mM sodium pyruvate, and 4.5% dextrose. The cells were grown in 25 cm^2^ culture flasks in the upright position in 10 mL aliquots of the culture medium. Incubation was performed at 37°C under a humidified atmosphere of 5% CO_2_-95% air.

### siRNA and transfection

The short interfering RNAs (siRNAs) were synthesized (GenePharma, China) and used for transfection. The sense and antisense strands of the ROCK-1 siRNA were 5′-GGCAGAGGAAGAAUAUAAATT −3′ and 5′- UUUAUAUUCUUCCUCUGCCTT-3′; ROCK-2 siRNA were 5′-GCAGCUGGAAUCUAACAAUTT-3′ and 5′- AUUGUUAGAUUCCAGCUGCTT-3′; negative control were 5′-UUCUCCGAACGUGUCACGUTT-3′ and 5′-ACGUGACACGUUCGGAGAATT-3′. (designed and synthesized by GenePharma Co. Ltd, Shanghai). These siRNA were transfected into Y79 cells with Lipofectamine® 2000 (Invitrogen) following the manufacturer’s instructions. Briefly, a total of 5 × 10^5^ cells were plated in 6-well plates and transfected using 100 pmol siRNA and 5 μL of Lipofectamine® 2000 per well. After 24–48 hours of incubation, the cells were harvested for RT-qPCR or western blots analysis.

### RNA isolation and RT-qPCR

Y79 cells transfected with the siRNA were incubated for 24 hours. Cells were harvested for RT-qPCR analysis. Total RNA was extracted using TRIzol (Invitrogen), and single-stranded cDNA was synthesized with AMV Reverse Transcriptase System (Promega) according to the manufacturer’s instructions. Real-time quantitative polymerase chain (RT-qPCR) reactions were done with 10 ng cDNA in SYBR Green I mix (Takara Bio Inc.) and run on an ABI Prism 7300 HT 7300 Real-Time PCR System (Applied Biosystems). For all of cDNA, 40 cycles and annealing temperature of 60°C (31 seconds) were used. All PCR reactions were performed in triplicate. Primer sequences (designed by primer3 web (version 3.0.0) and synthesized by Sangon Biotech Co. Ltd, Shanghai) are:

ROCK-1: forward primer 5′-ACCTGTAACCCAAGGAGATGTG-3′ and reverse primer 5′- CACAATTGGCAGGAAAGTGG-3′;

ROCK-2: forward primer 5′-AAGTGGGTTAGTCGGTTG-3′ and reverse primer 5′-GGCAGTTAGCTAGGTTTG-3′;

β-actin: forward primer 5′-GGGACCTGACTGACTACCTCA-3′ and reverse primer 5′-GACTCGTCATACTCCTGCTTG-3′. Commercial software (SDS version 1.3; ABI) was used to calculate 2^-ΔΔCt relative expression values for ROCK-1 and ROCK-2 normalized to the β-actin endogenous control.

### Western blots

Y79 cells transfected with the siRNA were incubated for 48 hours. Cells were harvested for western blots analysis. Y79 cells were lysed for 5 minutes in cold lysis buffer. After centrifugation at 12,000 rpm for 5 minutes, the supernatant was collected as the total cellular protein extracts. The protein concentration was determined using Bradford method [15]. The total cellular protein extracts were separated on 8% SDS–PAGE. Proteins were electrotransferred to PVDF membranes (Millipore, USA) by a semi-dry transferor. The membranes were blocked in 5% skimmed milk in TBS-T containing 0.05% Tween 20 at room temperature (RT) for 2 hours, and then incubated at RT for 2 hours with antibodies to ROCK-1, ROCK-2, LIMK2, phospho-LIMK1 Thr508/LIMK2 Thr505, cofilin, phospho-cofilin Ser3 (1:1000, Cell Signaling Technology, Beverly, MA), and β-actin(1:5000, Cell Signaling Technology, Beverly, MA) diluted in 5% bovine serum albumin in TBS-T, respectively, followed by incubating with the appropriate HRP-linked secondary antibodies. Finally, the bands of specific proteins on the membranes were developed with Western Blotting Luminal Reagent (Millipore, USA) and quantified with Image J software (National Institutes of Health, USA).

### Cell proliferation assay

Cell proliferation was detected by a Cell Counting Kit-8 (Beyotime, Jiangsu, China) assay. Y79 cells were suspended in RPMI 1640 medium supplemented with 15% heat-inactivated fetal bovine serum and subsequently seeded in 96-well plates and incubated for 24 h. After that, we added medium containing Y-27632 ((R)-(+)-trans-N-(4-Pyridyl)-4-(1-aminoethyl)- cyclohexanecarboxamide.2HCl, purchased from Enzo Life Sciences, Inc.) in 10, 50, 100 μM, respectively for 48 hours. Then the cultures were added 10 μl CCK-8 solution to each well and incubated at 37°C for another 2 hours. Optical density (OD) value of absorbance at 450 nm was measured by Thermo Scientific Fluoroskan Ascent FL (Thermo Fisher Scientific Inc.). The results were plotted as means ± SD of three independent experiments having three determinations per sample for each experiment.

### Cell adhesion assay

The effects of Y-27632 on the adhesion ability of Y79 cells to ECM were examined using the adhesion assay. The binding of Y79 cells to matrigel were investigated. A 96-well plate was coated with matrigel (2 ug/50 μl). Cells (4 × 10^5^ cells, cultured in RPMI 1640 medium supplemented with 10% FBS) were seeded onto these components in 96-well Plate. Y-27632 was diluted at the concentration of 10 μM, 20 μM, 30 μM, 50 μM, 100 μM. The cells were treated for 1 h. They were allowed to adhere to each well for 30 min at 37°C and then gently washed twice in PBS. The adhesion Y79 cells were quantified by the Cell Counting Kit-8 assay according to the manufacturer’s instructions.

To study the effects of ROCK siRNA on cell adhesion, three groups of Y79 cells were transfected for 24 hr with control siRNA, ROCK-1 siRNA, or ROCK-2 siRNA, respectively. The cells in the forth group were treated with Y-27632 at 30 *μ*M. Untransfected cells and cells transfected with control siRNA served as controls. Cells (4 × 10^5^ cells) were allowed to adhere to each well for 30 min at 37°C and then gently washed twice in PBS. The adhesion Y79 cells were quantified by the Cell Counting Kit-8 assay.

### Cell invasion assay

The 24-well plate Transwell system with a polycarbonate filter membrane of 8-mm pore size (Corning Inc.) was used to detect invasive capacity changes of Y79 cells. The Matrigel (BD Biosciences) layers were rehydrated for 2 hours at RT by adding 100 μL of serum-free media to the upper compartment. After rehydration, the media were removed from the upper compartment of the invasion chamber. Cell suspension containing 1 × 10^6^ cells/mL was prepared in serum-free media, 400 μl RPMI 1640 medium supplemented with 15% FBS were added to each of the lower compartments and 100 μL of the cell suspension were added to each of the upper compartments. After incubating for 48 hours at 37°C, invasive cells in lower compartments of the chamber were counted by Cell Counting Kit-8 assay.

The six groups of Y79 cells were treated with Y-27632 at 0, 10 *μ*M, 20 *μ*M, 30 *μ*M, 40 *μ*M, 50 *μ*M, respectively. After incubating for 48 h at 37°C, invasive cells in lower compartments of the chamber were counted by Cell Counting Kit-8 assay. To study the effects of ROCK siRNA on cell invasion, three groups of Y79 cells were transfected for 24 hr with control siRNA, ROCK-1 siRNA, or ROCK-2 siRNA, respectively. The cells in the forth group were treated with Y-27632 at 30 *μ*M. Untransfected cells and cells transfected with control siRNA served as controls. After incubating for 48 h, invasive cells in lower compartments of the chamber were counted by Cell Counting Kit-8 assay.

### Statistical analyses

All experiments were repeated at least three times. Independent t-test analysis was used for statistical analysis between two groups, and the comparisons among multiple groups were made with a one-way analysis of variance (ANOVA) followed by Dunnett’s t test. (SPSS Statisics 19.0). The differences were considered significant for *P* values of < 0.05.

## Result

### Expressions of ROCK-1 and ROCK-2 in Y79 cell lines

ROCK-1 and ROCK-2 mRNA expression in Y79 cell lines were examined by RT-qPCR.

**Figure 1:**
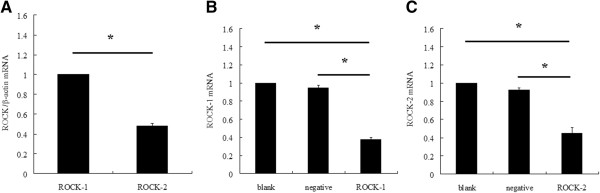
**ROCK-1 and ROCK-2 mRNA expression in Y79 cell lines. (A)** ROCK-1 and ROCK-2 mRNA expression in Y79 cell (*P < 0.05, n = 3). **(B)** ROCK-1 mRNA level in Y79 cell in blank control group, negtive control group and ROCK-1 siRNA group (*P < 0.05, n = 3). **(C)** ROCK-2 mRNA level in Y79 cell in blank control group, negtive control group and ROCK-2 siRNA group (*P < 0.05, n = 3).

The expression of mRNA of ROCK-1 was about twice as much as that of ROCK-2 ( 2^-ΔΔCt = 0.48 ± 0.03, *P* < 0.05) (Figure 1A). Treatment of Y79 cells with ROCK-1 siRNA for 24 hours reduced ROCK-1 mRNA levels against blank controls (2^-ΔΔCt = 0.38 ± 0.03, *P* < 0.05) and negative controls (2^-ΔΔCt = 0.40 ± 0.02, *P* < 0.05) (Figure 1B). Treatment of Y79 cells with ROCK-2 siRNA for 24 hours reduced ROCK-2 mRNA levels against blank controls (2^-ΔΔCt = 0.45 ± 0.05, *P* < 0.05) and negative controls (2^-ΔΔCt = 0.48 ± 0.05, *P* < 0.05) (Figure 1C).

**Figure 2:**
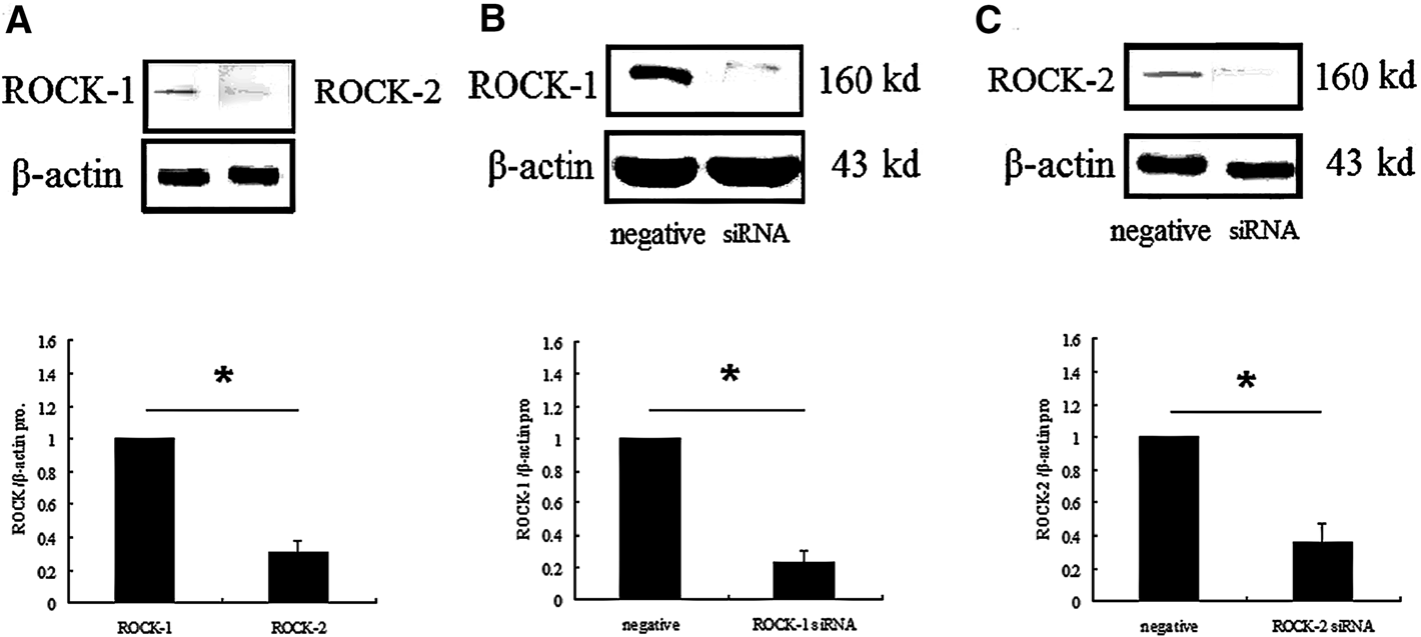
**ROCK-1 and ROCK-2 protein expression in Y79 cell lines. (A)** ROCK-1 and ROCK-2 protein expression in Y79 cell (*P < 0.05, n = 3). **(B)** Western blotting shows the marked decrease in the band intensity of the Y79 cells treated with ROCK-1 siRNA for 48 hours compared to negative control siRNA treatment (*P < 0.05, n = 3). **(C)** Western blotting shows the marked decrease in the band intensity of the Y79 cells treated with ROCK-2 siRNA for 48 hours compared to negative control siRNA treatment (*P < 0.05, n = 3).

Protein expression in Y79 cell line that received siRNA treatment for ROCK-1 and ROCK-2 were examined by using western blot analyses. Cells transfected with control siRNA were used as controls. The protein expression levels of ROCK-1 and ROCK-2 were reduced in the cells transfected with siRNA against ROCK-1 and ROCK-2, respectively, compared with the control cells (Figure 2).

### Effects of Y-27632 on cell proliferation of Y79 cells

**Figure 3:**
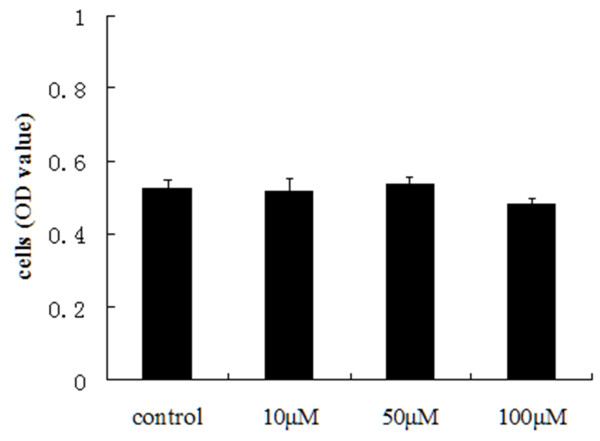
**Effects of Y-27632 concentration on cell of Y79 cell proliferation.** The OD value of CCK-8 assay shows no difference in cell proliferation of Y79 cells at various Y-27632 doses (*P < 0.05, n = 5).

To investigate the effects of ROCK inhibition on cellular proliferation of Rb cells, cell proliferation was evaluated by CCK-8 assay after exposure to various Y-27632 doses. Y-27632 treatment didn’t show any changes of Y79 cells proliferation (*P >* 0.05) (Figure 3). These results showed that ROCK inhibitor Y-27632 didn’t influence proliferation of Rb cell in vitro.

### Effects of Y-27632 and ROCK siRNA on adhesion capacity of Y79 cells

**Figure 4:**
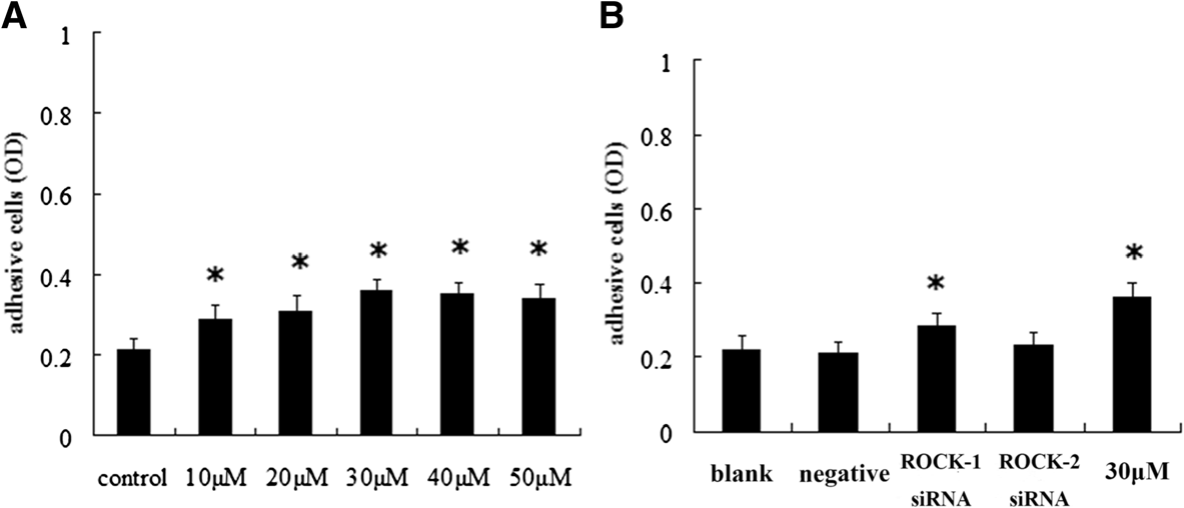
**Effects of Y-27632 and ROCK siRNA on adhesion capacity of Y79 cells. (A)** Effects of Y-27632 on adhesion capacity of Y79 cells. Compared with the control group, adhesion capacity increased with increasing Y-27632 concentration (*P < 0.05, n = 3). **(B)** Adhesion capacity of the cells treated with blank control, negative control siRNA, ROCK-1 siRNA, ROCK-1 siRNA, and y-27632 at 30 *μ*M (*P < 0.05, n = 3).

The Y-27632 dosage-dependent effect on Y79 cell adhesion capacity was studied. The adhesion capacity showed a positive correlation with increasing Y-27632 concentration (Figure 4A). The OD value of cells transfected with ROCK-1 siRNA were increased, comparing with blank and negative control group (*P* < 0.05) (Figure 4B), but it was similar to cells treated with Y-27632 at 30 *μ*M (*P* > 0.05). There was no statistical difference between the cells transfected with ROCK-2 siRNA and negative control group (*P* > 0.05).

### Effects of Y-27632 and ROCK siRNA on invasive capacity of Y79 cells

**Figure 5:**
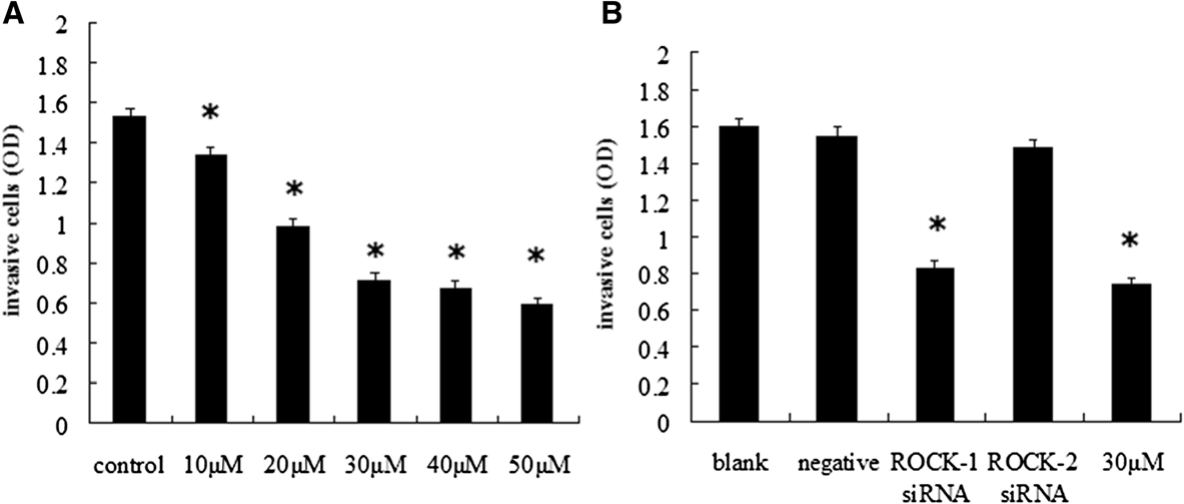
**Effects of Y-27632 and ROCK siRNA on invasive capacity of Y79 cells. (A)** Effects of Y-27632 on invasion capacity of Y79 cells. Compared with the control group, invasion capacity decreased with increasing Y-27632 concentration (*P < 0.05, n = 3). **(B)** Invasion capacity of the cells treated with blank control, negative control siRNA, ROCK-1 siRNA, ROCK-1 siRNA, and y-27632 at 30 *μ*M (*P < 0.05, n = 3).

The Y-27632 dosage-dependent effect on Y79 cell invasion capacity was studied. After 48 hours incubation at 37°C, the invasion capacity showed an inverse relationship with increasing Y-27632 concentration (Figure 5A). The OD value of cells transfected with ROCK-1 siRNA were decreased, comparing with blank and negative control group (*P* < 0.05) (Figure 5B), but it was similar to cells treated with Y-27632 at 30 *μ*M (*P* > 0.05). There was no statistical difference between the cells transfected with ROCK-2 siRNA and negative control group (*P* > 0.05).

### Y-27632 and ROCK siRNA inhibits the phosphorylation of LIMK2 and Cofilin

**Figure 6:**
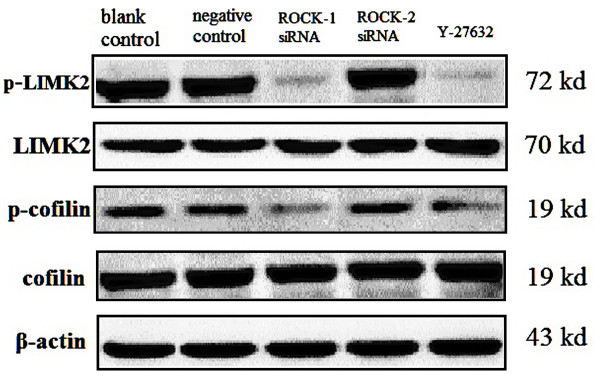
**Y-27632 or ROCK-1 siRNA inhibits LIMK2/cofilin pathway.** Effects of Y-27632 and ROCK siRNA on the ROCK/LIMK2/cofilin signaling pathway by Western blot analysis, Y-27632 or ROCK-1 siRNA could inhibit the levels of LIMK2 and cofilin phosphorylation in Y79 cells (n = 3).

We investigated the effects of Y-27632 and ROCK siRNA on the ROCK/LIMK2/cofilin signaling pathway by Western blot analysis. As we expected, Y-27632 or ROCK-1 siRNA could inhibit the levels of LIMK2 and cofilin phosphorylation in Y79 cells (Figure 6).

## Discussion

ROCK-1 and ROCK-2 are serine/threonine kinases and work as effectors of RhoA, a small GTPase [16]. RhoA/ROCK pathway has been shown to work multifunctionally in various cell behaviors [6]. ROCK-1 is expressed ubiquitously, while ROCK-2 is expressed mainly in the brain, muscle, heart, lung, and placenta [17]. In this study, we found that ROCK-1 and ROCK-2 were expressed in Y79 cell lines at both mRNA and protein levels. The expression of mRNA of ROCK-1 was almost twice as much as that of ROCK-2. The expression of ROCK-1 and ROCK-2 were downregulated in the cells transfected with siRNA with ROCK-1 and ROCK-2 mRNA inhibition.

ROCK-1 and ROCK-2 are the main downstream effectors of Ras homologous (Rho) family of GTPases. ROCK phosphorylates the conserved threonine in the activation loops of LIM kinase-2 (LIMK2), increasing LIMK activity and the subsequent phosphorylation of cofilin proteins, which blocks their F-actin-severing activity [18-20]. ROCK activation leads to a series of events that promote force generation and morphological changes. These events contribute directly to a number of actin-myosin mediated processes, such as cell motility, adhesion, smooth muscle contraction, neurite retraction and phagocytosis. In addition, ROCK kinases play roles in proliferation, differentiation, apoptosis and oncogenic transformation, although these responses can be cell type-dependent [7,21]. Y-27632 can inhibit both ROCK-1 and ROCK-2. In the present study, proliferation of Y79 cells was not altered significantly after treatment of Y-27632.

Furthermore, ROCKs regulate cell migration in part by enhancing actomyosin contractility. ROCK activity has been reported to be required for tail retraction, at least in monocytes and prostate cancer cells [22,23].

**Figure 7:**
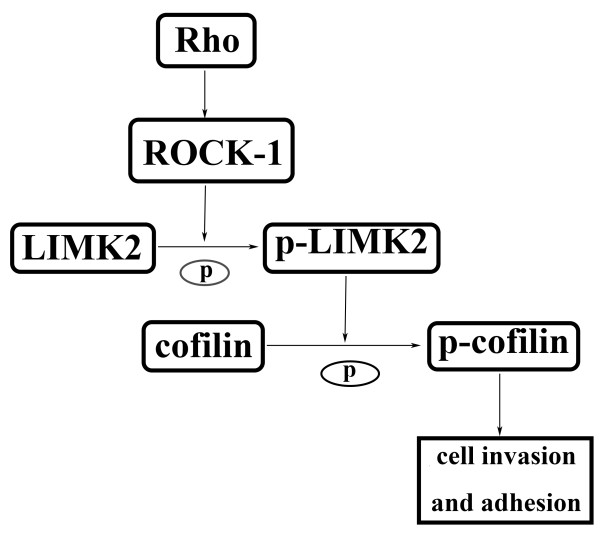
**Rho/ROCK-1/LIMK2/cofilin signalling passway.** ROCK-1 is downstream effector of Rho. ROCK-1 phosphorylates and activates LIM-kinase, which in turn phosphorylates and inactivates cofilin, and that regulates cell adhesion and invasion.

ROCKs can also affect cell migration by limiting the extent of lamellipodial protrusion, as inhibiting ROCK or Rho induces the occurrence of membrane ruffles [24]. In addition, ROCK-mediated activation of LIMKs and subsequent cofilin phosphorylation could affect cell migration [25], as constitutively active LIMK2 or excess phosphorylated cofilin inhibit cell polarization by inducing the formation of numerous lamellipodia (Figure 7) [25]. The cell invasion assay using transwell chambers showed that invasion ability was decreased in Y79 cells transfected with ROCK-1 siRNA, and the invasion ability could also be decreased by Y-27632, however, invasion ability was not decreased in Y79 cells transfected with siRNA against ROCK-2, indicating that ROCK-1 but not ROCK-2 is involved in invasion ability of Y79 cells. We hypothesized that the reason was lower expression of ROCK-2 than that of ROCK-1 in Y79 cells.

Taken together, the findings of this study demonstrate that cell mobility and invasive ablity of Rb could be decreased by ROCK-1-inhabitor, Therefore, the ROCK-1-dependent pathway may be a potential target for therapy of Rb.

## Conclusions

In conclusion, results described in this report suggest that ROCK-1 protein plays a key role in regulating metastasis and invasion of Y79 cells, Therefore, ROCK-1 dependent pathway may be a potential target for therapy of Rb.

## Competing interests

The authors declare that they have no competing interests.

## Authors’ contributions

JW, YSZ, and BX conceived and designed the experiments. JW, XHL, and ZJY performed the experiments. JW, XHL, and ZJY analyzed the data. JW, YSZ, BX, XHL, and ZJY wrote the paper. All authors read and approved the final manuscript.

## Pre-publication history

The pre-publication history for this paper can be accessed here:

http://www.biomedcentral.com/1471-2407/14/89/prepub

## References

[B1] Jabbour P Chalouhi N Tjoumakaris S Gonzalez LF Dumont AS Chitale R Rosenwasser R Bianciotto CG Shields C Pearls and pitfalls of intraarterial chemotherapy for retinoblastoma J Neurosurg Pediatr 2012 10 3 175 181 10.3171/2012.5.PEDS1277 22793160

[B2] Abramson DH Retinoblastoma: diagnosis and management CA Cancer J Clin 1982 32 3 130 140 10.3322/canjclin.32.3.130 6804033

[B3] Lawler K Foran E O’Sullivan G Long A Kenny D Mobility and invasiveness of metastatic esophageal cancer are potentiated by shear stress in a ROCK- and Ras-dependent manner Am J Physiol Cell Physiol 2006 291 4 C668 C677 10.1152/ajpcell.00626.2005 16641163

[B4] Schofield AV Steel R Bernard O Rho-associated Coiled-coil Kinase (ROCK) protein controls microtubule dynamics in a novel signaling pathway that regulates cell migration J Biol Chem 2012 287 52 43620 43629 10.1074/jbc.M112.394965 23093407 PMC3527948

[B5] Nakagawa H Yoshioka K Miyahara E Fukushima Y Tamura M Itoh K Intrathecal administration of Y-27632, a specific Rho-associated kinase inhibitor, for rat neoplastic meningitis Mol Cancer Res 2005 3 8 425 433 10.1158/1541-7786.MCR-05-0002 16123138

[B6] Riento K Ridley AJ Rocks: multifunctional kinases in cell behaviour Nat Rev Mol Cell Biol 2003 4 6 446 456 10.1038/nrm1128 12778124

[B7] Shimada T Nishimura Y Nishiuma T Rikitake Y Hirase T Yokoyama M Adenoviral transfer of rho family proteins to lung cancer cells ameliorates cell proliferation and motility and increases apoptotic change Kobe J Med Sci 2007 53 3 125 134 17684444

[B8] Ro¨sel D Bra’bek J Tolde O Mierke CT Zitterbart DP Raupach C Bicanova’ K Kollmannsberger P Pankova’ D Vesely P Up-regulation of Rho/ROCK signaling in sarcoma cells drives invasion and increased generation of protrusive forces Mol Cancer Res 2008 6 9 1410 1420 10.1158/1541-7786.MCR-07-2174 18819929

[B9] Xue F Takahara T Yata Y Xia Q Nonome K Shinno E Kanayama M Takahara S Sugiyama T Blockade of Rho/Rho-associated coiled coil-forming kinase signaling can prevent progression of hepatocellular carcinoma in matrix metalloproteinase-dependent manner Hepatol Res 2008 38 8 810 817 10.1111/j.1872-034X.2008.00333.x 18507693

[B10] Vishnubhotla R Sun S Huq J Bulic M Ramesh A Guzman G Cho M Glover SC ROCK-II mediates colon cancer invasion via regulation of MMP-2 and MMP-13 at the site of invadopodia as revealed by multiphoton imaging Lab Invest 2007 87 11 1149 1158 10.1038/labinvest.3700674 17876296

[B11] Lane J Martin TA Watkins G Mansel RE Jiang WG The expression and prognostic value of ROCK I and ROCK II and their role in human breast cancer Int J Oncol 2008 33 3 585 593 18695890

[B12] Ishizaki T Uehata M Tamechika I Keel J Nonomura K Maekawa M Narumiya S Pharmacological properties of Y-27632, a specific inhibitor of rho-associated kinases Mol Pharmacol 2000 57 5 976 983 10779382

[B13] Routhier A Astuccio M Lahey D Monfredo N Johnson A Callahan W Partington A Fellows K Ouellette L Zhidro S Pharmacological inhibition of Rho-kinase signaling with Y-27632 blocks melanoma tumor growth Oncol Rep 2010 23 3 861 867 20127030

[B14] Itoh K Yoshioka K Akedo H Uehata M Ishizaki T Narumiya S An essential part for Rho-associated kinase in the transcellular invasion of tumor cells Nat Med 1999 5 2 221 225 10.1038/5587 9930872

[B15] Kruger NJ The Bradford method for protein quantitation Methods Mol Biol 1994 32 9 15 7951753 10.1385/0-89603-268-X:9

[B16] Ishizaki T Maekawa M Fujisawa K Okawa K Iwamatsu A Fujita A Watanabe N Saito Y Kakizuka A Morii N The small GTP-binding protein Rho binds to and activates a 160 kDa Ser/Thr protein kinase homologous to myotonic dystrophy kinase EMBO J 1996 15 8 1885 1893 8617235 PMC450107

[B17] Nakagawa O Fujisawa K Ishizaki T Saito Y Nakao K Narumiya S ROCK-I and ROCK-II, two isoforms of Rho-associated coiled-coil forming protein serine/threonine kinase in mice FEBS Lett 1996 392 2 189 193 10.1016/0014-5793(96)00811-3 8772201

[B18] Scott RW Olson MF LIM. kinases: function, regulation and association with human disease J Mol Med 2007 85 6 555 568 10.1007/s00109-007-0165-6 17294230

[B19] Ohashi K Nagata K Maekawa M Ishizaki T Narumiya S Mizuno K Rho-associated kinase ROCK activates LIM-kinase 1 by phosphorylation at threonine 508 within the activation loop J Biol Chem 2000 275 5 3577 3582 10.1074/jbc.275.5.3577 10652353

[B20] Sumi T Matsumoto K Nakamura T Specific activation of LIM kinase 2 via phosphorylation of threonine 505 by ROCK, a Rho-dependent protein kinase J Biol Chem 2001 276 1 670 676 11018042 10.1074/jbc.M007074200

[B21] Michael Olson F Applications for ROCK kinase inhibition Curr Opin Cell Biol 2008 20 2 242 248 10.1016/j.ceb.2008.01.002 18282695 PMC2377343

[B22] Worthylake RA Lemoine S Watson JM Burridge K RhoA is required for monocyte tail retraction during transendothelial migration J Cell Biol 2001 154 1 147 160 10.1083/jcb.200103048 11448997 PMC2196864

[B23] Somlyo AV Bradshaw D Ramos S Murphy C Myers CE Somlyo AP Rho-kinase inhibitor retards migration and in vivo dissemination of human prostate cancer cells Biochem Biophys Res Commun 2000 269 3 652 659 10.1006/bbrc.2000.2343 10720471

[B24] Worthylake RA Burridge K RhoA and ROCK promote migration by limiting membrane protrusions J Biol Chem 2003 278 15 13578 13584 10.1074/jbc.M211584200 12574166

[B25] Dawe HR Minamide LS Bamburg JR Cramer LP ADF/cofilin controls cell polarity during fibroblast migration Curr Biol 2003 13 3 252 257 10.1016/S0960-9822(03)00040-X 12573223

